# Preparation of polycaprolactone nanoparticles via supercritical carbon dioxide extraction of emulsions

**DOI:** 10.1007/s13346-017-0422-3

**Published:** 2017-08-21

**Authors:** Adejumoke Lara Ajiboye, Vivek Trivedi, John C. Mitchell

**Affiliations:** Faculty of Engineering and Science, University of Greenwich, Central Avenue, Chatham Maritime, Kent, ME4 4TB UK

**Keywords:** Solvent extraction, Polycaprolactone, Nanoparticles, Supercritical carbon dioxide, Supercritical fluid extraction of emulsions

## Abstract

Polycaprolactone (PCL) nanoparticles were produced via supercritical fluid extraction of emulsions (SFEE) using supercritical carbon dioxide (scCO_2_). The efficiency of the scCO_2_ extraction was investigated and compared to that of solvent extraction at atmospheric pressure. The effects of process parameters including polymer concentration (0.6–10% *w*/*w* in acetone), surfactant concentration (0.07 and 0.14% *w*/*w*) and polymer-to-surfactant weight ratio (1:1–16:1 *w*/*w*) on the particle size and surface morphology were also investigated. Spherical PCL nanoparticles with mean particle sizes between 190 and 350 nm were obtained depending on the polymer concentration, which was the most important factor where increase in the particle size was directly related to total polymer content in the formulation. Nanoparticles produced were analysed using dynamic light scattering and scanning electron microscopy. The results indicated that SFEE can be applied for the preparation of PCL nanoparticles without agglomeration and in a comparatively short duration of only 1 h.

## Introduction

The use of drug delivery systems, particularly the micro- and nano-scale intelligent systems for therapeutic molecules has rapidly increased over the years because they can successfully maximise the efficacy of the drug molecules [1]. Nanoparticles are particles with a diameter range from 1 to 100 nm, even though the dynamic range can cover the whole nanometre scale [2]. As a drug delivery system, nanoparticles can entrap drugs or biomolecules into their internal structures and/or adsorb these drugs or biomolecules onto their external surfaces [3].

Due to their particle size, nanoparticles can move freely through the body via the smallest capillary vessels, cell and tissue gaps in order to reach their target organs [4]. These properties of nanoparticles help to modify the bio-distribution and pharmacokinetic properties of the adsorbed/entrapped drug molecules leading to an improvement in the efficacy of the drug, decrease in unwanted side-effects and increase in patient compliance [5]. Nanoparticles can be prepared from inorganic or polymeric materials. Polymeric nanoparticles are more common and appropriate as they can be chemically modified to be biodegradable and biocompatible [6]. Biodegradable substances are broken down in vivo either enzymatically or non-enzymatically or both, to give rise to toxicologically safe by-products which are further removed by the normal metabolic pathways. The use of biodegradable polymers has greatly increased over the past decade, and they can generally be classified as either (1) synthetic biodegradable polymers which include relatively hydrophobic materials for example poly-lactic-co-glycolic acid (PLGA), polycaprolactone (PCL) and others, or (2) naturally occurring polymers such as chitosan, hyaluronan, etc. [7]. Principally, synthetic polymers have many inherent advantages since their structures can be manipulated to generate specialised carriers to suit particular applications [8].

PCL is a semi-crystalline polyester that is hydrophobic, biodegradable and biocompatible. When compared to other polymers, the biodegradation of PCL is slow; hence, it can be highly suitable for the design of controlled release delivery systems [9–11]. The glass transition temperature (*T*_g_) of − 60 °C and low melting point (59–64 °C) of PCL allows for the easy fabrication of delivery systems at reasonably low temperatures [10]. Furthermore, PCL has an excellent blend compatibility with other polymers which facilitates tailoring of desired properties like degradation kinetics, hydrophilicity and mucoadhesion [12, 13].

In the last decades, PCL polymers have been a key area of interest in the development of controlled delivery systems especially for peptides and proteins [14]. These systems using PCL or PCL blends have shown to be useful in varied conditions. For example, while developing PCL microspheres for vaccine delivery, Jameela et al. showed that PCL has good permeability to proteins and contrary to poly (lactic acid) PLA and poly glycolic acid (PGA), PCL degrades very slowly and does not give rise to an acidic environment which can negatively affect the antigenicity of the vaccine, and thus, it can be used as a vaccine carrier [15]. Observations by Youan et al. also suggested that PCL delivery systems are unaffected by simulated gastric fluid and hence could give some protection to the encapsulated peptide from proteolytic destruction in the stomach, allowing the entrapped drugs to pass unharmed into the intestine for presentation to the gut-associated lymphoid system [16].

Various techniques can be employed to prepare nanoparticles, many of which involve a type of emulsion evaporation. The preparation of nano-emulsions via spontaneous emulsification mechanism using a water-miscible solvent is an established method that allows for the generation of nano-sized droplets [17]. Nano-emulsions are made of fine oil-in-water dispersions with droplet sizes ranging from 100 to 600 nm. They can be prepared using a small surfactant concentration and they typically exhibit low oil-water interfacial tensions. Spontaneous emulsification occurring when the organic and aqueous phases are in contact determines the droplet size and size distribution, and depends on variables including bulk viscosity, surfactant concentration and structure [17–19]. In this work, the organic phase consists of a homogenous solution of the polymer in a water-miscible solvent (acetone) and surfactant which is mixed with the aqueous phase to produce nano-emulsion.

Although emulsification of polymer mixtures is known to be an efficient method, it can be associated with limitations concerning process efficiency and control of the particle size and distribution when particles are being recovered from the emulsion through conventional processes [20]. Supercritical fluid extraction of emulsion (SFEE) is a novel particle formation technique where it employs a supercritical fluid (SCF) such as supercritical carbon dioxide (scCO_2_) to rapidly extract the solvent or oil phase of an emulsion. The removal of the solvent leads to the precipitation of the solute resulting into an aqueous suspension containing nanoparticles. Particles can thereafter be recovered from the aqueous suspension by centrifugation and/or by evaporation at room temperature [21]. Particles generated via SFEE have an ordered size and morphology because emulsification process provides a template and the fast kinetics of the scCO_2_ extraction prevents particle agglomeration [22].

The aim of this work was to produce polymeric nanoparticles by employing scCO_2_ extraction of the solvent in the organic phase of nano-emulsions prepared via spontaneous emulsification. The major objective of this work was to study the suitability of SFEE and the effect of various parameters influencing the preparation of nanoparticles using PCL as a model polymer. The effect of parameters including polymer and surfactant concentration on the particles’ hydrodynamic diameter, polydispersity index (PDI) and zeta potential was investigated. The time and the optimum CO_2_ flow rate were also determined for the complete extraction of oil phase/solvent at both high pressures in scCO_2_ and atmospheric conditions.

## Experimental section

### Materials

PCL (Mw = 45,000 Da) was obtained from Shenzhen ESUN, China, and Tween 80 and acetone were purchased from Sigma Aldrich, UK. Liquid CO_2_ at 99.9% was supplied by BOC Ltd., UK. All chemicals were of analytical grade and used without any further purification. Deionised water was used throughout the study.

### Methods

#### Oil-in-water emulsion preparation

The nano-emulsions were prepared through spontaneous emulsification mechanism. The method used for the preparation of PCL emulsions was adapted from the work reported by Prieto and Calvo [22]. The organic phase consists of a homogenous solution of PCL in acetone and Tween 80, while deionised water made up the aqueous phase. In summary, an appropriate quantity of PCL was first dissolved in acetone (0.6–10% *w*/*w*) in a thermostatic water bath at 40 °C, before the addition of surfactant in a centrifuge tube to obtain required concentrations (0.07 and 0.14%) or ratios (1:1–16:1 *w*/*w*). The contents in the tube were mixed together using a vortex mixer for 1 min. After vortexing, 35.8 g of warm deionised water (40 °C) was added, and the tube was placed back on the vortex mixer for 5 min in order to form a homogenous emulsion. Solvent extraction was carried out immediately after emulsion was prepared.

#### Solvent extraction via SFEE

SFEE was conducted in a batch mode where 25-g emulsion was introduced in the high-pressure vessel (Thar Process Inc., USA) pre-heated to 40 °C (± 2 °C), the vessel was then closed and liquid CO_2_ was pumped until a pressure of 100 bar was achieved. The system was left to equilibrate under stirring for 15 min before continuously flushing with fresh CO_2_ for 1 h. The initial experiments were carried out at controlled scCO_2_ flow rates of 1, 4 or 10 g/min to determine the extraction efficiency of scCO_2_. The system was then depressurised at a rate of 6 bar/min, and the sample was removed from the high-pressure vessel. The acetone extraction efficiency was calculated using the weight difference before and after the procedure.

#### Solvent extraction at atmospheric pressure

Solvent extraction at atmospheric pressure was conducted at 40 °C (± 2 °C) in a thermostatic water bath (Fisher Scientific, UK) with 25-g emulsion in a 50-ml beaker, at a stirring rate of 300 rpm. The evaporation of acetone was determined at time 0, 1, 2, 3, 4 and 24 h using weight difference similar to SFEE.

### Physicochemical characterisation of nanoparticle

#### Particle size and zeta potential measurement

These measurements were carried out in order to determine the size and surface charge of the nanoparticles. The average particle sizes and polydispersity index (PDI) were measured by dynamic light scattering (DLS) using a Zetasizer Nano-ZS (Malvern Instruments Ltd., UK). The instrument was equipped with a laser emitting at 633 nm, and backscattering detection was set at an angle of 173°. Samples were diluted in deionised water (1 in 4) and measurements were carried out in triplicate at room temperature (25 °C). The mean particle size was the average of three independent measurements. The zeta potential (*Z*-potential) of the aqueous dispersions was also determined using Zetasizer Nano-ZS at 25 °C. The measurements were carried out in triplicate on the same samples used for particle size measurements.

#### Particle morphology

Scanning electron microscopy (SEM) was performed in order to determine the shape and surface morphology of the nanoparticles. The frozen aqueous nanoparticle dispersions were freeze-dried under vacuum at − 55 °C using a ScanVac CoolSafe freeze dryer (LaboGene ApS, Denmark). Approximately 1 mg of the dried particles was placed on the sample stub using a carbon adhesive and the extra loose particles were removed. The sample was then coated with chromium, thereafter the stub was mounted onto the sample holder and placed inside the Hitachi SU8030 (Hitachi High-Technologies, UK) scanning electron microscope. Micrographs were collected using the upper detector at a voltage of 1.0 kV.

## Results and discussion

### Solvent extraction

**Fig. 1:**
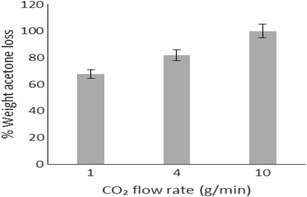
Acetone extraction via SFEE

The rate of acetone extraction via supercritical processing was evaluated. The temperature (40 °C) and pressure (100 bar) selected for SFEE were based on the low melting point (59–64 °C) of the PCL polymer and the good miscibility of acetone with scCO_2_ at these conditions [23]. Figure 1 shows that at a constant exposure time of 1 h, the percentage weight of acetone extracted from the emulsion increases with an increasing flow rate of scCO_2_. Complete removal of acetone occurred after 1 h at a CO_2_ flow rate of 10 g/min. Extraction of the organic phase can occur either by diffusion mechanism in the aqueous phase or by direct extraction upon contact between acetone and scCO_2_ in the emulsion droplet. Thus, acetone extraction at higher scCO_2_ flow rates improves as it allows for a comparatively large quantity of CO_2_ to be in contact with the emulsion and consequently the organic phase [24].

**Fig. 2:**
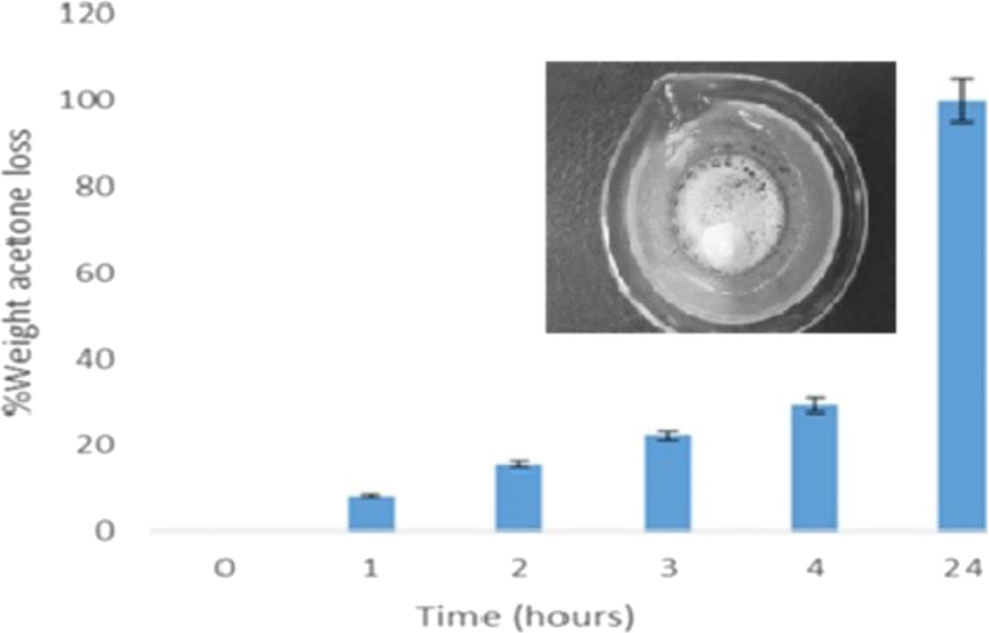
Acetone extraction at atmospheric pressure. *Inset*: an image of typical agglomerate seen after acetone extraction at 40 °C

The rate of acetone extraction at atmospheric conditions was also evaluated and compared to scCO_2_ conditions. Acetone extraction at atmospheric pressure was carried out at 40 °C to obtain comparative data at the same temperature used in supercritical fluid extraction. Also, the conditions used in these experiments allowed the work to be carried at a temperature below the melting point of PCL (59–64 °C) to avoid possible particle deformation due to simultaneous melting of the polymer. The complete removal of acetone at atmospheric pressure (Fig. 2) could only be obtained after 24 h of continuous stirring at 40 °C.

Moreover, the extraction at atmospheric pressure also led to the agglomeration of particles (Fig. 2, inset) consequently decreasing the total product yield. The gas-like viscosity of scCO_2_ coupled with high diffusivity facilitates the rapid and efficient extraction of acetone in comparison to solvent extraction at atmospheric pressure at the same temperature without particle agglomeration [25]. Based on these findings (rapid extraction time and no aggregation at scCO_2_ conditions), SFEE was chosen as the method for solvent removal for the rest of the study. The extraction conditions were kept constant with a scCO_2_ flow rate of 10 g/min for 1 h at 100 bar and 40 °C.

### Effect of polymer concentration

PCL content was varied between 0.6 and 10% *w*/*w*, and the effects of polymer concentration on the morphological characteristics and surface charge of the particles were investigated.

**Fig. 3:**
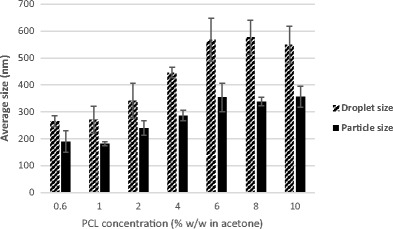
The effect of PCL concentration in the organic phase on the average sizes before and after SFEE

As noted in Fig. 3, at a constant surfactant concentration (0.07% *w*/*w*) and water-to-acetone ratio (2.5:1), an increase in polymer concentration results in an increase in particle size. These results are in line with findings of Santos et al. [26] and Kwon et al. [27]. Primarily, it is assumed that nanoparticle formation occurs when both the organic and aqueous phases are in contact. The solvent diffuses from the organic part into the aqueous and takes along with it some polymer chains which are still in solution, then as the solvent spreads further into the water, the accompanying polymer chains aggregate forming nanoparticles [28]. A rise in the polymer content in the organic phase consequently increases both its hydrophobic composition and viscosity which is relative to a higher mass transfer resistance, hence leading to a negative effect on the distribution efficiency of the polymer-solvent composition into the external aqueous phase and the formation of large nanoparticles [27, 28]. The results also show a marked difference between the size before and after acetone extraction, which could be due to the presence of voids in the polymer matrix which collapse upon the extraction of acetone by scCO_2_. It is well known that free spaces within the polymer network caused by the presence of a solvent can cause particles to shrink and reduce in size after drying [29].

The particle surface charge was determined by carrying out *Z*-potential measurements. Table 1 presents the *Z*-potential values of PCL nanoparticles prepared with different polymer concentrations. Nanoparticles produced have a negative surface charge which can be as a result of the carbonyl group of the PCL polymer present at the surface of the nanoparticle structure. Table 1 also shows a shift in *Z*-potential values with an increasing polymer concentration. The change in *Z*-potential can be attributed to the shielding effect by the surfactant molecules at the interface due to interaction with PCL. Therefore, as the polymer content increases at a constant surfactant concentration, the number of Tween 80 molecules available at the interface decreases consequently resulting in a reduced shielding effect and leading to a change in *Z*-potential [30]. Table 1 also presents PDI of produced nanoparticles at various polymer concentrations. In general, the PDI was very low for all formulations, confirming that the prepared nanoparticles are highly monodisperse and have narrow particle size distribution. A slight increase in PDI was observed with the increase in polymer concentration and particle size. This could be attributed to the increase in viscosity of the organic phase and possible coalescence of smaller droplets into larger droplets at higher concentrations resulting in comparatively broad particle size distribution [31, 32].

**Table 1:** PDI and *Z*-potential of PCL particles before and after extraction of a 25-g emulsion (0.6–10% *w*/*w* PCL, 28.3% *w*/*w* of acetone, 71.6% *w*/*w* of distilled water and 0.07% *w*/*w* surfactant)

PCL concentration (% *w*/*w* in acetone)	PDI ± SD before SFEE	PDI ± SD after SFEE	*Z*-potential (mV) ± SD after SFEE
0.6	0.13 ± 0.07	0.11 ± 0.10	−10 ± 1
1	0.19 ± 0.06	0.09 ± 0.07	−12 ± 1
2	0.09 ± 0.07	0.16 ± 0.08	−15 ± 2
4	0.17 ± 0.04	0.22 ± 0.04	−24 ± 1
6	0.23 ± 0.07	0.27 ± 0.04	−21 ± 0
8	0.21 ± 0.05	0.27 ± 0.02	−24 ± 1
10	0.21 ± 0.04	0.25 ± 0.02	−26 ± 1

### Effect of surfactant concentration

In order to investigate the effect of surfactant on the particle size, PDI and surface charge, at a constant polymer concentration, the surfactant content was doubled from 0.07 to 0.14% *w*/*w*.

**Fig. 4:**
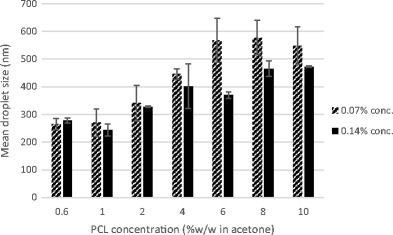
Comparison of the mean emulsion droplet size (nm) of 0.07 and 0.14% *w*/*w* surfactant concentrations at a constant PCL concentration (% *w*/*w* in acetone)

As observed in Fig. 4, there is a general increase in droplet size as polymer concentration increases. Furthermore, an increase in surfactant concentration resulted in smaller droplet size which was more evident at higher polymer concentrations. This result is similar to the findings from the work done by Goloub and Pugh [33]. The positive effect of a higher Tween 80 concentration on droplet size can be easily related to the role of Tween 80 in the emulsion. A low concentration of surfactant in the emulsion and consequently at the solvent-water interfacial layer would mean that there is not enough surfactant to cover the surface of drops, resulting in coalescence of droplets and increase in size. An increase in the surfactant concentration would therefore result in a higher number of surfactant molecules present at the interfacial layer promoting the formation of smaller droplets [34].

**Fig. 5:**
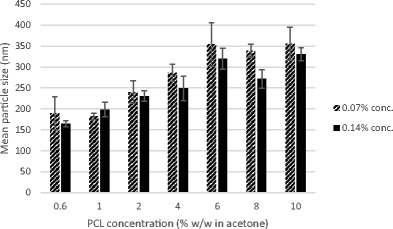
Contrast of the mean particle size (nm) of 0.07 and 0.14% *w*/*w* surfactant concentrations after SFEE at a constant PCL concentration (% *w*/*w* in acetone)

Similar to earlier observations, an increase in polymer concentration at a constant surfactant concentration generally resulted in an increase in particle size. As noted when Figs. 4 and 5 are compared, there is also a reduction in size after acetone extraction by SFEE.

Figure 5 also shows that a higher surfactant concentration led to decrease in particle size and standard error. This result is similar to that reported by Mu and Feng [35]. The reduction in size can also be anticipated from the role of surfactant as an emulsion stabiliser in the formation of nanoparticles [36].

*Z*-potential measurements (Table 2) were determined on 0.14% *w*/*w* Tween 80 samples to understand the effect of the surfactant concentration on the particle surface charge. Similar to earlier results, *Z*-potential increased with the polymer concentration but no significant change could be observed with the higher surfactant content in these samples.

**Table 2:** PDI and *Z*-potential of PCL nanoparticles before and after SFEE of a 25-g emulsion (0.6–10% *w*/*w*, 28.3% *w*/*w* of acetone, 71.6% *w*/*w* of distilled water and 0.14% *w*/*w* surfactant)

PCL concentration (% *w*/*w* in acetone)	PDI ± SD before SFEE	PDI ± SD after SFEE	*Z*-potential (mV) ± SD after SFEE
0.6	0.09 ± 0.03	0.11 ± 0.05	−14 ± 1
1	0.14 ± 0.05	0.19 ± 0.04	−17 ± 3
2	0.09 ± 0.04	0.14 ± 0.07	−19 ± 3
4	0.14 ± 0.08	0.19 ± 0.08	−22 ± 2
6	0.16 ± 0.06	0.22 ± 0.12	−25 ± 3
8	0.19 ± 0.04	0.26 ± 0.09	−29 ± 3
10	0.19 ± 0.06	0.24 ± 0.05	−29 ± 3

Like the previous results, the PDI recorded for the higher surfactant concentration samples (Table 2) are very low for all the preparations and they indicate that the nanoparticles produced have a uniform particle size distribution.

### Effect of polymer-to-surfactant weight ratio

To further study the effect of varying surfactant/polymer content on nanoparticle formation, the concentrations were altered to prepare emulsions with polymer-to-surfactant weight ratios of 1:1, 2:1, 4:1, 8:1 and 16:1.

**Fig. 6:**
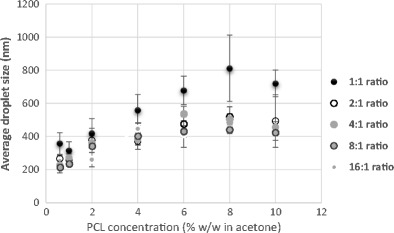
Mean emulsion droplet size at a varying polymer-to-Tween 80 ratio (1:1–16:1) and a constant PCL concentration

**Fig. 7:**
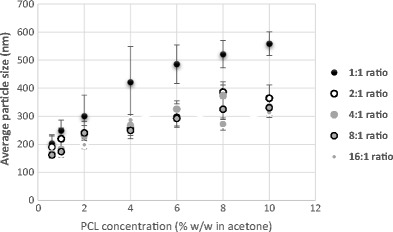
Mean particle size after SFEE at a varying polymer-to-Tween 80 ratio (1:1–16:1) and a constant PCL concentration

As shown in Figs. 6 and 7, similar to previously discussed findings, at a constant polymer-to-surfactant weight ratio, increasing polymer concentration led to the formation of larger particles. Interestingly, 1:1 *w*/*w* ratio samples have noticeably higher particle size in comparison to the rest of the data sets. This could be attributed to the depletion effect from non-adsorbing Tween 80 micelles at such high surfactant concentration [37]. The presence of free micelles at high surfactant concentrations can cause increase in osmotic pressure in the liquid surrounding the particles to an extent which can result in particle aggregation [38].

It is also important to note that the error associated to the particles prepared with 1:1 *w*/*w* ratio is also very high in comparison to other formulations which suggest flocculation/aggregation of prepared particles. There were minimal changes in particle sizes at polymer-to-surfactant weight ratio from 16:1 to 2:1 *w*/*w*. These results suggest that it may be possible to prepare PCL nanoparticles in wide ranging sizes using extremely high surfactant-to-polymer ratio but factors mentioned above are important to be considered. However, particle sizes ranging from 150 to 350 nm with narrow distribution can be reproducibly obtained using low to moderately high surfactant concentrations.

### Particle morphology

**Fig. 8:**
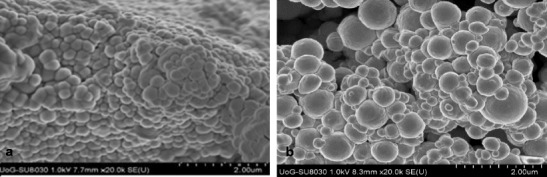
Example of SEM micrographs of particles processed by SFEE. **a** 0.6% *w*/*w*. **b** 10% *w*/*w* at ×20,000 magnification

SEM micrographs were obtained using chromium-coated freeze-dried PCL nanoparticles in order to investigate the shape and surface morphology of produced particles. Figure 8 presents an example of micrographs of PCL nanoparticles prepared by SFEE.

From the SEM micrographs, it is clearly seen that the particles prepared by SFEE are spherical and generally with uniform particle size distribution. SEM micrographs also confirm DLS results that there is an increase in particle size at a higher polymer concentration.

## Conclusion

Spherically shaped monodisperse polycaprolactone nanoparticles have been successfully prepared by supercritical fluid extraction of emulsions without agglomeration and in a comparatively short duration. Varying the polymer concentration between 0.6 and 10% *w*/*w* during the formulation stage allowed for a control of the particle size of the resulting polycaprolactone nanoparticles. An increase in the emulsion surfactant concentration from 0.07 to 0.14% *w*/*w* at a constant polymer concentration led to a decrease in particle size. There was minimal change in size when the polymer-to-surfactant weight ratio was varied between 2:1 and 16:1 *w*/*w* ratios. 1:1 *w*/*w* polymer-surfactant weight ratio produced polycaprolactone nanoparticles with sizes between 200 and 500 nm with high standard error between repeats.

Although preliminary studies have shown that these polycaprolactone nanoparticles can be efficiently produced via supercritical fluid extraction of emulsions, further studies investigating their potential to be used as delivery vehicles either by surface adsorption of a drug or encapsulation within to provide modified release need to be carried out.
